# An overview of current advances in perinatal alcohol exposure and pathogenesis of fetal alcohol spectrum disorders

**DOI:** 10.1186/s11689-024-09537-w

**Published:** 2024-04-20

**Authors:** Xingdong Zeng, Yongle Cai, Mengyan Wu, Haonan Chen, Miao Sun, Hao Yang

**Affiliations:** 1 Institute for Fetology, The First Affiliated Hospital of Soochow University, Suzhou, 215031 China; 2 School of Basic Medical Sciences, Ningxia Medical University, Yinchuan, 750004 Ningxia China

**Keywords:** Prenatal alcohol exposure, Alcohol spectrum disorder, Fetal alcohol syndrome, Pathogenesis, Neuroinflammation, Neurotoxicity

## Abstract

The adverse use of alcohol is a serious global public health problem. Maternal alcohol consumption during pregnancy usually causes prenatal alcohol exposure (PAE) in the developing fetus, leading to a spectrum of disorders known as fetal alcohol spectrum disorders (FASD) and even fetal alcohol syndrome (FAS) throughout the lifelong sufferers. The prevalence of FASD is approximately 7.7 per 1,000 worldwide, and is even higher in developed regions. Generally, Ethanol in alcoholic beverages can impair embryonic neurological development through multiple pathways leading to FASD. Among them, the leading mechanism of FASDs is attributed to ethanol-induced neuroinflammatory damage to the central nervous system (CNS). Although the underlying molecular mechanisms remain unclear, the remaining multiple pathological mechanisms is likely due to the neurotoxic damage of ethanol and the resultant neuronal loss. Regardless of the molecular pathway, the ultimate outcome of the developing CNS exposed to ethanol is almost always the destruction and apoptosis of neurons, which leads to the reduction of neurons and further the development of FASD. In this review, we systematically summarize the current research progress on the pathogenesis of FASD, which hopefully provides new insights into differential early diagnosis, treatment and prevention for patents with FASD.

## Introduction

Alcohol abuse is a global problem, currently shows an upward trend in both the amount and rate of alcohol consumption per capita worldwide [1]. Almost any level of alcohol consumption is considered harmful, however, it is almost impossible to achieve this global goal of reducing alcohol consumption [1, 2]. More strikingly, the global rate of alcohol consumption among women during pregnancy is also increasing, and has hitherto reached 9.8% [3]. Accordingly, prenatal alcohol exposure (PAE) is also the most common cause of fetal origin disease [4].

Fetal Alcohol Spectrum Disorder (FASD) is a general term for multiple manifestations of fetal defects that is likely caused by PAE [5, 6]. Once aggravated with permanent neurological and birth defects, FASD will develop into FAS [5, 6]. Due to direct, lasting, multisystemic and irreversible damage to the embryo, the global prevalence of FASD is approximately 7.7 per 1000 population (95% CI, 4.9–11.7 per 1000 population), and varies by different regions [7, 8]. For instance, the developed regions such as Europe have the highest prevalence (19.8 per 1,000) and the Mediterranean region has the lowest prevalence (0.1 per 1,000) [8]. In a survey of mainstream elementary school in Manchester, UK, the crude prevalence of FASD reached an alarming 1.8% (95% CI: 1.0%, 3.4%) [9]. Based on a systematic review of 23,470 studies from Svetlana Popova, approximately 15% women who drink during pregnancy will give birth to children with Fetal Alcohol Syndrome (FAS) [3].

Although there are a variety of prenatal exposures which could cause embryonic neurodevelopmental disorders, in terms of exposure risk and degree of nerve damage, alcohol is more severe than other risky substances like tobacco and marijuana [10]. PAE often affects the neurological development of the embryo, resulting in FASD and even FAS, which is indicative of the most typical features, such as morphological changes, cognitive impairment, and behavioral deficit. Regarding abnormalities of these aspects, a study done by Amanda Facciol showed the aforementioned abnormalities in a zebrafish model with different alcohol gradients [11]. Notably, PAE remarkably affected genotypes, displaying a significant increase in anxiety-like behavior [11]. In addition, a systematic evaluation and comprehensive analysis of patients has demonstrated a causal relationship between PAE and fetal cognitive, learning, and memory deficits [12]. This implied that alcohol has a significant negative impact on neurodevelopment, which is proportional to the severity of PAE [13]. Concurrently, the complicated medical and social obstacles associated with FAS in children severely compromise their physical and mental development. A meta-analysis from Canada indicated that 428 comorbidities co-occur with FASD, which includes genetic, homeostatic, behavioral disorders and so on [14, 15]. Additionally, social issues such as dating, schooling, and maintaining stable employment will follow [16]. Given that the onset of FASD is mostly silent and undetectable and the underlying mechanism is still unknown, it is challenging to reduce the harmful use of alcohol for decrease of frequency of occurrence or presence of FASD. Presently, it has been shown that maternal alcohol consumption during pregnancy causes FASD, and chronic alcohol consumption by the father also causes FASD (This mainly ascribes to the effect of alcohol on the embryo through the father’s spermatogenesis) [17, 18]. Therefore, a better understanding of the molecular mechanisms underlying FASD is very important to prevent and treat FASD. Furthermore, it is imperative to explore effective treatment strategies for FASD and give patients with FASD some special cares at the societal level to reduce stigma, improve quality of life and prevent the occurrence of FASD in future generations [1, 14, 16, 19]. The multiple hazards brought by the aforementioned PAE and the challenge for treatment of FASD all imply an urgent need for immediate and efficient action to resolve this serious issue [20].

Owing to a serious disruption of PAE in embryo's brain development and the complexity of the pathogenesis, diagnosis and treatment FASD, a further understand the pathophysiology and pathogenesis of FASD is vital to develop optimal strategies to prevent, diagnose, and achieve breakthroughs of treatment of FASD. This review will focus mainly on the molecular mechanism of FASD pathogenesis, treatment, prevention and future research breakthroughs.

The search of cited research was conducted in electronic databases from dates April 2023: PubMed, MEDLINE, Embase, Web of Science Core Collection, Cochrane Database of Systematic Reviews. Title and abstract screening were performed in two stages by one reviewer, supported by a second reviewer. Full‐text screening, data extraction, and quality appraisal were performed by two reviewers independently. The papers selected were English language only.

### Neuronal damage mechanism of FASD

Among a host of embryonic diseases, prenatal alcohol exposure (PAE) is the most frequent cause of a variety of non-genetic factors resulting in fetal neurodevelopmental or fetal neuro-related behavioral abnormalities. Alcohol could directly or indirectly impair development of embryos in utero in a multi-systemic, multi-faceted, and multi-level manner, leading to the development of FASD in the fetus after birth, or further developing into FAS [4]. The main mechanism of FASD is closely related with alcohol-induced neuroinflammation and oxidative stress, by which potentially result in neural cell apoptosis in the developing embryonic brain, thus impeding the development of the embryonic brain [7, 21]. Ethanol neurotoxicity is another major mechanism by which ethanol causes FASD by direct damage to various signaling pathways [7, 22]. Through several signal transduction pathways, including G protein-coupled receptor, tyrosine kinase receptor and insulin receptor, ethanol can subtly interfere with the normal development of the embryonic nervous system [23, 24]. In addition, through epigenetic mechanisms like aberrant DNA methylation and gene expression (transcription, translation, etc.), excessive alcohol use also impedes the neurological system development [7, 25, 26]. Although ethanol can cause extensive damage to normal tissues and cells, the exact mechanisms of alcohol neurotoxicity remain unknown. Therefore, more research needed to be further investigated.

In spite of alcohol consumed by the mother, the main composition of alcohol (ethanol and its metabolite acetaldehyde) has the ability to cross the placental barrier and directly harm an embryo's developing nervous system. The alcohol-Induced damage to the developing nervous system is associated with ethanol-induced oxidative stress and neurotoxicity which elicit varying degrees of neural cell apoptosis, degeneration or necrosis, eventually resulting in neurodevelopmental abnormalities and functional deficits [21, 22, 27]. Regarding this point, more PAE animal models and studies of children with FASD both demonstrate this standpoint [28, 29]. In short, ethanol could affect the development of nerve cells through various signaling pathways, eventually leading to a decrease of total numbers of neurons in the embryonic nervous system and the resultant neural dysfunction and neurodevelopmental defects in children.

### Oxidative stress injury

The pathogenesis of FASD is mainly attributed to the autophagy of growing neurons following the inflammatory response caused by ethanol in the fetal body through oxidative stress, which is directly linked to the raise in reactive oxygen species (ROS) in the embryo after ethanol ingestion [21, 22]. It is reported that apart from reactive oxygen and nitrogen species, ROS includes at least one oxygen atom and one or more unpaired electrons, can live independently in the human body [30]. The group is generally composed of oxygen radicals, including hydroxyl, singlet oxygen, hydrogen peroxide, free nitrogen, and superoxide anion radicals. Consequently, ROS are the generic term for a group of substances [31]. Previously, ROS were considered as toxic byproducts of the body's oxygen metabolism, which could damage DNA directly or indirectly before causing cell death [32, 33]. However, with in-depth understanding of ROS, a growing number of studies showed that a particular biological level of ROS is constantly generated in the human body. As a signal molecule in numerous regular physiological processes of the human body, ROS are primarily produced by mitochondria and NADPH oxidase (NOX) in cells [30, 34, 35]. Each ROS has distinct biochemical properties and participates in various bodily physiological processes [31, 34, 35]. Once ROS level reaches physiological threshold concentration, it can function as anti-microbial effectors and signal molecules, and interacts with the protein complex nuclear transcription factor-kappa β (NF-β), which controls DNA transcription [34]. Notably, the body regular metabolism is mainly due to the balance between generation and removal of free radicals, which is conducive to natural selection through regulation of physiological differentiation and death of healthy cells [30, 31, 35]. However, once the equilibrium between antioxidant and oxidative factors or the generation and removal of ROS are disturbed, it will cause oxidative stress to harm the human body [36]. For instance, a long-term alcohol abuser’s body usually has a variety of oxidation/anti-oxidation imbalances [37]. In the research of PAE mice, the increased expression of different NOX subunits was found to result in excessive ROS production [38]. In addition, reduced expression of antioxidant enzymes such as superoxide dismutase, glutathione peroxidase and catalase were identified [39]. This is likely to due to an imbalance ROS in the developing embryo resulting in oxidative damage. Based on these reports, we speculate the ROS in the embryo and uterus are more likely to be elevated as maternal alcohol abuse during pregnancy through the placental barrier, and the increased ROS breaks the equilibrium [21, 40]. As a result, the embryo development of different system including the nervous system are usually attacked [21, 40, 41]. In general, the numerous systems that are affected by oxidative stress produce an imbalance of ROS, resulting in cell death, vascular sclerosis, an increase in autoimmune illnesses, and others [41]. As a result, damage to the developing nerve system in the womb causes FASD [21, 40]. The damage to DNA and the damage triggered by associated inflammatory factors serve as the primary pathogenesis of the process of ROS-induced oxidative stress leading to FASD [41]. This mechanism of damage is thus called as embryonic pathological neurogenesis.

**Fig. 1:**
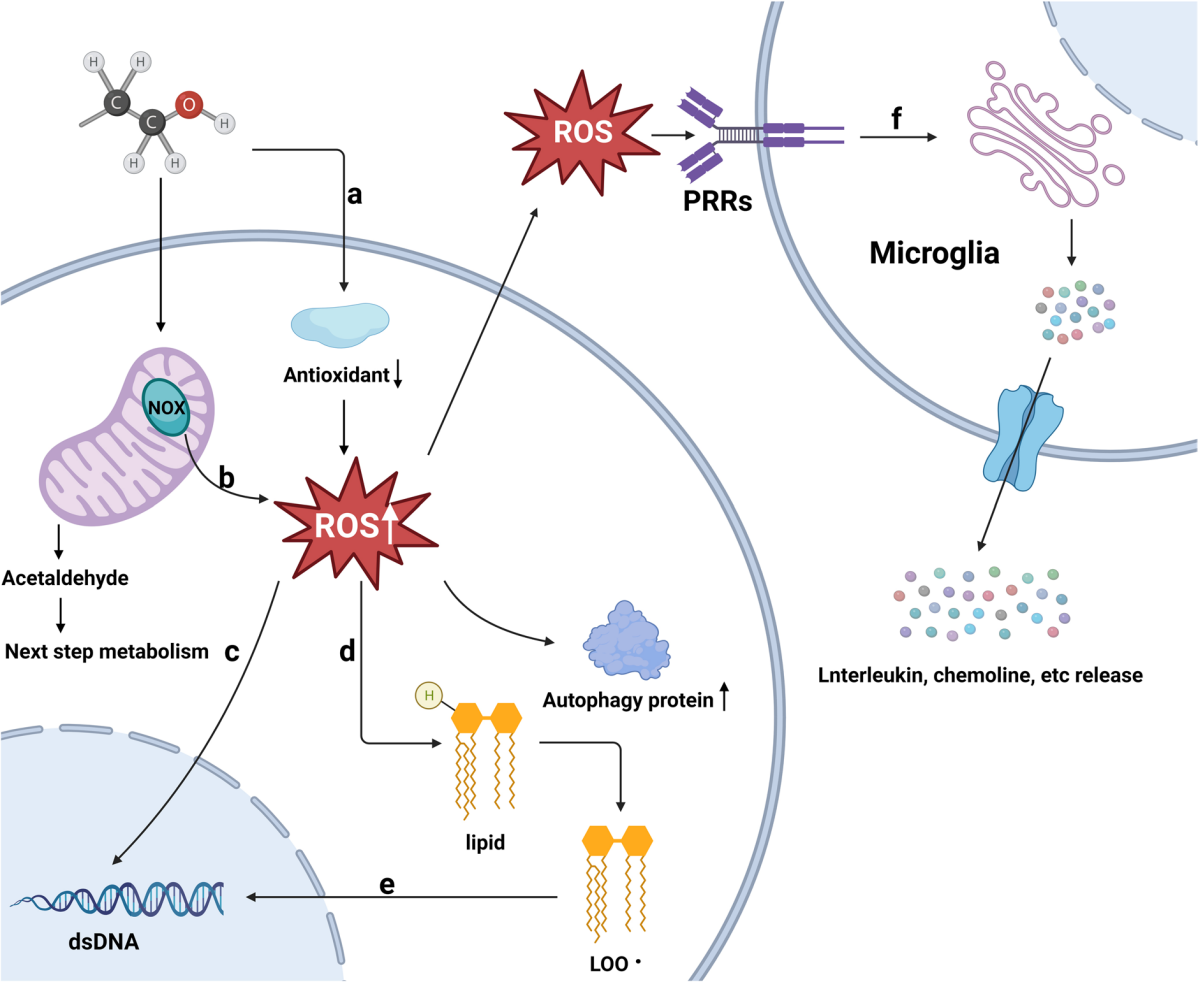
The damage of ROS from ethanol metabolism to neuronal development through multiple pathways. **a** Ethanol decreases the level of antioxidant enzymes in the embryo; **b** Ethanol is metabolized by NADPH oxidase in mitochondria to produce excessive by-products of ROS; **c** Overabundant ROS can enter the nuclei and directly attack dsDNA causing diverse types of DNA damage; **d** Overabundant ROS can catch H chain from lipids and peroxidize them to produce LOO; **e** The LOO ·produced bind to DNA to generate DNA adducts that indirectly damage dsDNA; **f** Overabundant ROS activates microglia via PRRs on the microglia membrane

The damage caused by oxidative stress cascade serves as the primary pathogenesis of the process of ROS-induced oxidative insult leading to FASD. In studies on C57BL/6 mice with PAE, both Jian Dong and Liya Qin found that the mRNA expression of the catalytic and regulatory subunit of NADPH oxidase (NOX) was significantly increased; however, after treatment with NOX enzyme inhibitor, ROS production and oxidative DNA damage in mice were significantly reduced. In addition, the incidence of embryonic cell was also significantly reduced [42, 43]. Consistently, another study by Mara José Pérez, Roco Loyola, et al. also found that ethanol-induced oxidative stress, mitochondrial dysfunction, and damage to synaptic vesicle activity were all considerably reduced in the mice after treated with apocytin, a NADPH inhibitor [44]. These results demonstrate that exogenous ROS produced by the metabolism of NOX in cells, can result in cell DNA damage, apoptosis, and other damages, are increased by ethanol consumption (Fig. 1).

### ROS damage to DNA

PAE also induces the generation of ROS. Concomitantly, ROS can result in oxidative stress, leading to DNA damage of cells and cognitive impairments in embryo. In the cell nucleus, chromatin DNA can be attacked by ROS, including DNA damage from direct attack and indirectly induced lipid peroxidation to produce lipid peroxyl radicals (LOO•) to further form multiple DNA adducts and thus cause cell damage [32, 33]. It was reported by Zhen Luo et al., that exogenous ROS were capable to eliciting the trophoblastic ectoderm cell cycle arrest in the S phase and G2/M phase, and increased the autophagic protein expression in the endoplasmic reticulum and the rate of ectoderm cell apoptosis [22, 45]. Nevertheless, the limitation of this study is not to precisely elucidate how ROS result in DNA damage. Subsequently, this question was addressed through a series of studies by Lutfiya Miller-Pinsler et al. They found that the most common exogenous ROS were verified as main source of the DNA damage, and the cognitive and behavioral deficits of the offspring of the CD-1 mouse model of PAE pretreatment with phenyl-N-tert-butyl nitrone (PBN; 40 mg/kg), a free radio spin trap which can reduce ROS, were dramatically reduced as compared to those of regular PAE animals [46]. Especially, PBN pretreatment decreased postnatal learning deficit (*p* < 0.01) and EtOH-induced DNA oxidation in the brains of children (*p* < 0.05) [46]. For substantiating the view, the oxyguanine glycosylase 1 (Ogg1) gene involved in DNA repair, was later knocked out. Encouragingly, the pathogenicity and level of 8-oxo-2'- deoxyguanosine (8-oxodGuo), a marker of DNA double-strand break, which is a major product of DNA oxidation and belongs to premutagenic damage, were both increased, indicating that serious DNA damage does exist in PAE mice [40, 47]. Based on the results, ROS arising from embryonic ethanol exposure will produce severe DNA damage of cells and cognitive impairments in children [40, 45–47].

Although accumulating studies have shown that ROS can damage DNA, it is still unknown about how ROS damage DNA. Currently, the majority of the DNA damage induced by ROS, such as 8-oxo-purine(8-oxo-Pu), depends on the attack of the most reactive free radical, the hydroxyl radical (HO•), which is one of multiple ROS produced by the metabolism of ethanol [48]. Additionally, the attack by HO is totally responsible for the development of 5', 8-cyclic purine (cPu) damage both in vitro and in vivo [48]. Only hydroxyl radical attack to 2'-deoxyribose units results in C5' radicals, which are then cyclized with the C8 position of the purine base to produce 5', 8-cyclic purine (cPu) damage [49]. The cPu arising from ROS is a tandem damage in the DNA damage category that will significantly impair the gene transcription, expression, and inheritance. This damage is obvious consequence of alcohol exposure [49, 50]. Of course, the attack of DNA by ROS is far more than cPu. By utilizing isotope labeling ROS, Chrysostomos and Chatilialoglu found 8-oxo-7,8-dihydro-2'-deoxypurine(8-oxo-Pu), 5-carboxamido-5-formamido-2-iminohydantoin, and other ROS attack sites [51, 52]. Moreover, the measurement of purine oxidation damage products indicated that the free radicals generated by the Fenton reaction also raised [51, 52] Analytical techniques like high performance liquid chromatography coupled with electrospray ionization tandem mass spectrometry were used to assess the oxidative modification of DNA [53]. It can be found that the neutral and alkaline free radicals produced after DNA damage by ROS and caused the oxidized DNA through a common degradation pathway [51, 53].

DNA damage caused by ROS has a structural patten as well. After quantifying the damaged purines, Michael A. Terzidis et al., studied the topological structure of DNA helix through ROS and found that the purine oxidation in dsDNA follows the order pattern line (L), open loop (OC) and superhelix (SC) [54]. In addition, the G-quadruplex rich in tandem guanine G sustains less damage than other topper [54]. Based on this finding, hydroxyl radical damage primarily affects the lower structure of dsDNA and has less impact on the upper structure. However, it needs to be identified whether other kinds of ROS also cause damage. Nonetheless, DNA oxidative damage can also be caused by ROS through a variety of patterns besides different sites to attack the DNA. Direct mononuclear base damage, tandem damage, and DNA adducts produced from hypochloric acid (HOCl) and hypobromic acid (HOBr) can all result from the oxidative alteration of dsDNA by ROS [21, 32, 33]. ROS-induced DNA oxidative damage is usually triggered via DNA cross-linking, intra-chain and inter-chain cross-linking, and DNA–protein cross-linking apart from tandem damage [53, 55]. A new DNA damage mechanism involved in the hydroxyl radical has been gradually unveiled in recent years. This includes the introduction of carbonate anion created during the damage process as a reaction intermediate, and the performance of gene regulation through the base excision repair pathway [52]. Additionally, ROS can damage DNA in numerous indirect ways, including by an interaction between DNA and a byproduct of lipid peroxidation that results in DNA extra-circular damage and by accelerating the impairment of a protein that helps repair damaged DNA, leading to the blockade of DNA healing after damage [21, 32, 33].

### ROS and inflammatory factors

In addition to direct damage to DNA, ROS production by alcohol triggers an oxidative stress response which suppresses the growth of neural cells through mediation of inflammatory-related cytokines and several pathways, leading to cell apoptosis and FASD [21, 34, 36]. Astrocytes and microglia are CNS-resident innate immune cells, which are able to initiate the embryo's innate immune response to invasion of exogenous pathogens [56]. Nevertheless, astrocytes and microglia are also activated by foreign substances like the alcohol-induced ROS in the nervous system, influencing the development of nervous system and resulting in FASD [57, 58]. In several studies in C57BL/6 J mice, activated microglia were identified to up-regulate the amount of pro-inflammatory factors and NOX, thus increasing the production of ROS [43, 58, 59]. The exacerbation of neuroinflammation in the embryo can be attributed to the promotion of one another by microglia and ROS.

Through the use of their pattern recognition receptors (PRRs), astrocytes and microglia recognize the damage-related molecular pattern (DAMP) of damaged host cells or the pathogen-related molecular pattern (PAMP) obtained from microbes, triggering inflammatory signal transduction and innate immunity [56, 60, 61]. The well-characterized receptor in the family of PRRs is the toll-like receptor (TLR) [56]. TLR is an intact membrane protein with an extracellular leucine-rich structural domain that, once activated by ROS, causes the release of a number of inflammatory factors by first activating transcription factors like AP-1 and NF-κB and then initiating a further signaling cascade through kinases [56, 61, 62]. The synergy between various TLRs, which also exists in the series of TLRs and other PRRs (such as retinoic acid-inducible gene I-like receptors and NOD-like families), increases the inflammatory pathological process caused by astrocytes [60, 63]. Numerous studies have demonstrated that TLR in the alcohol exposure models can stimulate the production of various inflammatory factors such chemokines, resulting in a wide range of negative effects [64–66]. Lídia Cantacorps and Silvia Alfonso-Loeches also found that several pro-inflammatory factors and effectors, such as nuclear factor-kappa B p65, NOD-like receptor protein 3, caspase-1, and interleukin-1, have increased in PAE animal models [65, 67]. Consistently, according to the studies from Mar a Pascual, L dia Cantacorps and others, TLR4 and TLR2 responses are identified to be crucial in the cognitive and neurodevelopmental impairments of kids caused by alcohol-induced neuroinflammation [65, 67, 68]. Moreover, these increased levels of chemokines and cytokines can be employed as biomarkers of the neuroimmune response to ethanol [66, 68].

Neuroinflammation arising from ROS by ethanol metabolism has far more influence on the nervous system. In the quantitative study of glial cells, neuroinflammation had an impact on several parameters in the offspring mouse model of PAE. For instance, Victoria M. Niedzwiedz-Massey found that the number of oligodendrocyte and its progenitor cells showed a varying degree of decrease [69]. Meantime, the study also revealed that the expression of the markers for oligodendrocyte precursor cells, premyelinating oligodendrocytes and mature myelinating oligodendrocytes significantly decreased as a result of further decline in cell quantity [67, 69]. This result indicated that alcohol intervention can cause the death of oligodendrocytes as well as myelin degradation and the loss of white matter [67, 69]. In addition, the exposure of alcohol reduced the number of synapses and microglial branches, and the levels of proteolipid protein (PLP) and myelin basic protein (MBP) which are necessary for myelin synthesis [64, 67, 70]. Moreover, neuroinflammation by ethanol has impact on nerve functions. Jorge Montesinos, et al., found that the cognitive function of the TLR4-KO PAE mouse offspring was remarkably improved, and the production of cytokines/chemokines in the progeny was reduced considerably, indicating that neuroinflammation as well as the damage to myelinated fibers and synapses have been alleviated [64, 71]. All above points indicated that alcohol-induced neuroinflammatory response plays crucial role in the etiology of FASD. Accordingly, the neuroinflammation by alcohol is a long-lasting and irreversible impairment of brain function in children with FASD [70].

Inflammatory variables including inflammatory chemokines play a role in the etiology of FASD apart from the neuroinflammatory damage mediated by the PLR family. Chemokines are a class of 8–10 kDa secretory proteins with chemotactic properties [72]. Generally, the chemokine family is responsible for the activation, adhesion, and migration of leukocytes and numerous other cell types. Chemokines are also widely present in large numbers of infiltrating lymphocytes of all histologic types including trophoblast cells in placenta during pregnancy [73–75], and play a pivotal role in the normal embryonic growth and development of the embryo along with maintenance of a conventional homeostatic balance [74–76]. However, when the steady-state chemokines such as CXCL12 are increased or variably transcribed due to any undesirable exogenous factors including alcohol, the progression of disease will be sped-up [74–76]. This pathogenesis is also present in many atypical chemokines including atypical chemokine receptor 3 (ACKR3) [76]. An increasing number of studies have shown that the CXCL12/CXCR4/CXCR7 axis of chemokines has a prominent role in a variety of pregnancy-related diseases, resulting in different biological activities and signaling activation [74, 75, 77]. Moreover, the disease progression is also enhanced by the up-regulation of ACKR3 [75, 76]. Besides, inflammatory chemokines, including monocyte chemoattractant protein-1 (CCL2) and neutrophil chemokine-12a (CXCL12), not only accelerate the inflammatory process and recruitment of inflammatory cells, but also affect the migration and growth of some developing neurons. The pro-inflammatory chemokine systems CXCL12/CXCR4 and CCL2/CCR2 and the corresponding mRNAs were found to be increased after alcohol stimulation in a variety of PAE animal models, as demonstrated by PCR and immunofluorescence [78–80]. On the basis of the studies of Patrick P Lowe, Caroline Morel, it assumes that the upregulation of chemokines is critical for the activation and recruitment of mononuclear macrophages and microglia in the embryonic CNS [80–82]. Together, the inflammatory signals could be mediated through orchestrating multiple CCL2/CCR2/JAK2 and CXAL12a/CXCR4b signaling axis, and thereby exert the corresponding effects in exacerbation of neuroinflammatory damage [82–85].

In the etiological study of FASD, the development of neuroinflammation is promoted by a variety of inflammatory factors, including the pattern recognition receptor TLR, chemokines, etc. Chemokines have been proven to play a significant role in a variety of physiological and pathological processes that occur in the human body, including metabolism, inflammation [86]. Along with homeostatic processes, inflammatory responses, and pathological circumstances, the chemokine family is in charge of the activation and infiltration of leukocytes, and other pathophysiologic events of various cell types. The normal presence of conventional homeostatic chemokines, which contribute to embryonic growth and development, hematopoiesis, and angiogenesis, play a significant role in the normal growth and development of the embryo [74–76]. Undeniably, neuroinflammation is an immediate, long-lasting and irreversible influence on the pathogenesis of children with FASD [70]. The summary of oxidative stress injury through multiple molecular mechanism in Table 1.

**Table 1:** The summary of neurotoxic injury through multiple molecular mechanisms

**Oxidative injury**	**Authors**	**Methods**		**Main findings**
**Directly attacking DNA**	Zhen Luo et Aaron M Fleming et. Chryssostomos Chatgilialoglu et	Flow cytometry and immunofluorescence were applied to pTc cell lines after exogenous ROS intervention to detect cell cycle and apoptotic proteins Spectroscopic detection of intermediates generated by ROS damage by using an ICCD camera, measurement of purine damage by stable isotope LC–MS/MS labelling of ROS	**Manifestations** **Sites**	ROS cause ectodermal cell cycle arrest at S and G2/M phases and increase autophagy protein expression ROS injury DNA through sites: 8-oxo-Pu, 5carboxamido-5-formamido-2iminohydantoin
	Michael A Terzidis et. Jean Cadet et	Gamma-irradiation experiments were performed on the intervened pUC19 plasmid with dsDNA samples. Quantification of purine lesions by stable isotope LCMS/MS labeling	**Pathway**	Damage caused by ROS to DNA: tandem damage, intra- and interstrand crosslinks, and DNA–protein crosslinks. Injury starts at lower structures (L > OC > SC)
**Neuroinflammation**	María Pascual et. Lídia Cantacorps, et. Silvia AlfonsoLoeches et. María Pascual et Cynthia J M Kane, et. Jorge Montesinos et	C57BL/6 wild-type and TLR4-KO pregnant mice were exposed daily by drinking at timed whole-gestation intervals with 10–20% ethanol solution (v/v), 5 g/kg after which TLRs were quantified in the F1 generation by electron microscopy, ELISA C57BL/6 J mice were exposed by gavage for the entire gestation period using 30% ethanol solution (v/v) at 4 g/kg. Quantitative analysis by immunohistochemistry, quantitative PCR	**Signal transduction** **Inflammatory factors**	TLRs on the glial cell surface are activated by ROS thereby trigger signaling cascades to stimulate the production of inflammatory factors and effects. Activated TLRs transmits inflammatory signals through transcription factors AP-1 and NF-κB The ACKR3, CXCL12/CXCR4/CXCR7 axis of chemokines has a prominent role in FASD, resulting in differential biological activity and signaling activation
	Guo-Qing Chang et Kinning Poon et Kai Zhang et	Oral exposure of rats to 2 g/kg of ethanol during early to mid-pregnancy. For the F1 generation of C57BL/6 J mice 4 days after birth 5 g/kg of ethanol saline solution (20%) was given subcutaneously four times. F1 generation progenitor neurons were cultured. Analysis of chemokines by combining electron microscopy and immunofluorescence methods		CXCL12/CXCR4, CCL2/CCR2, and the corresponding mRNAs are increased upon alcohol stimulation, which is essential for the activation and recruitment of monocyte macrophages and microglia in the embryonic CNS

### Neurotoxic injury

**Fig. 2:**
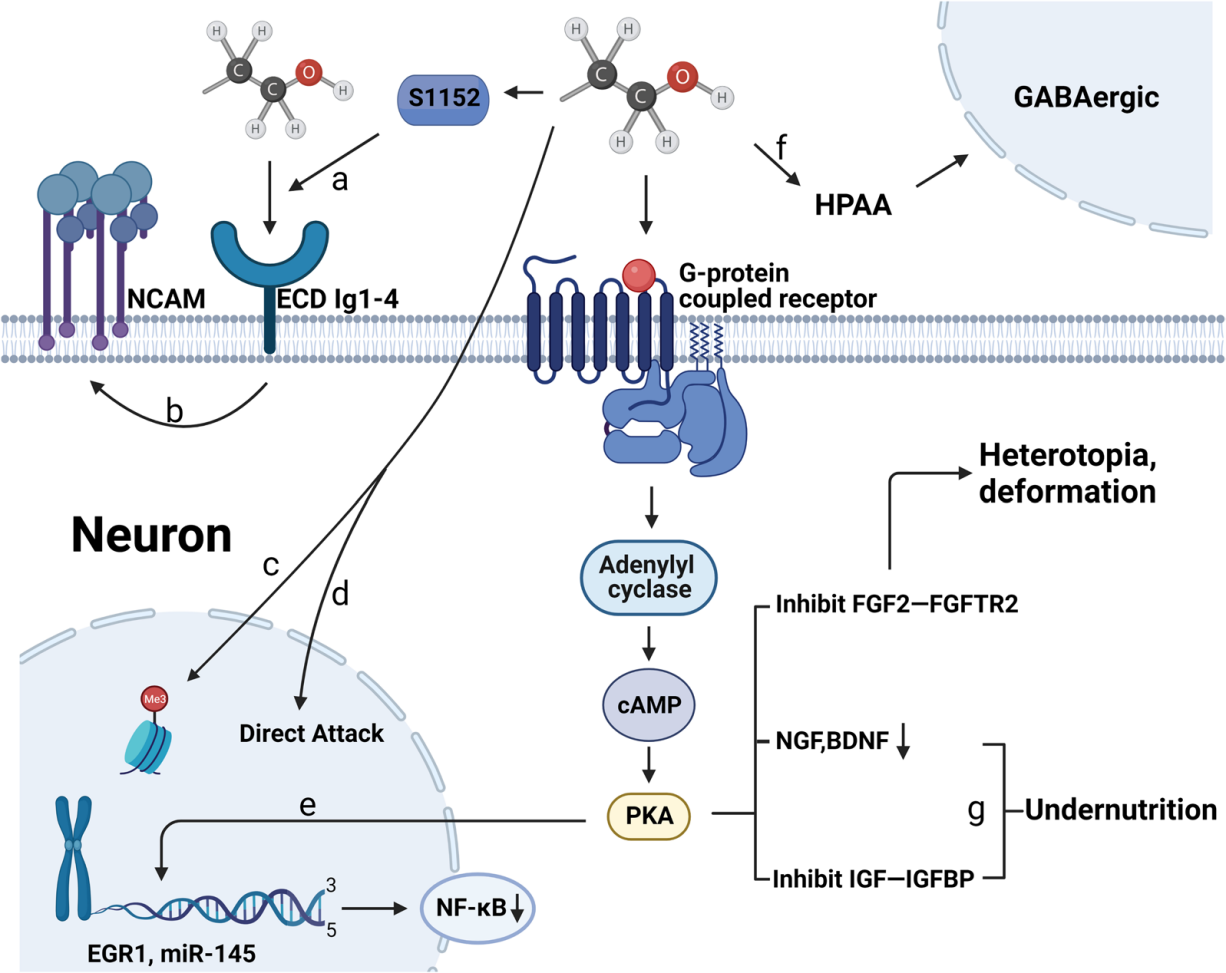
The FASD pathways of Ethanol-mediated neurotoxicity. **a** Ethanol causes an increase in the phosphorylation level of S1152, resulting in the structural domain ECD sensitive to ethanol more bound; **b** Ethanol binding ECD Ig-4 impairs the biofunction of NCAM; **c** Ethanol directly drives DNA methylation transferase to increase the methylation of genes; **d** Ethanol directly attacks DNA and its histones, causing cell cycle arrest and DNA breakage; **e** Ethanol causes a decrease in NF-κB through the cAMP/PKA pathway by targeting the promoter EGR1,miR-145, etc.; **f** Ethanol can hypersensitize the HPAA axis and consequently increase GABAergic interneuron; **g** Ethanol inhibits the IGF-IGFBP pathway and decreases the levels of NGF, BDNF, resulting in neuronal undernutrition and dysplasia

Another pathogenesis of alcoholic damage is mainly attributed to neurotoxic effects of ethanol on embryonic nerve cells leading to FASD. Nevertheless, the specific mechanism of ethanol neurotoxicity in current studies is still not clarified. In fact, ethanolic neurotoxic damage to the embryo is a multifactor interdynamic, complicated dynamic processes which usually involves a wide range of potential events, such as alteration of brain structure, aberrant activation of multiple signaling cascades in cells, disruption of intracellular homeostasis (e.g. Ca^2+^), abnormal expression of multiple genes, and autophagy in developing cells [7, 22, 29]. Apart from neuroinflammatory ROS damage, almost all other pathogenic mechanisms can be summarized as neurotoxic damage from ethanol (Fig. 2).

### Epigenetic damage of ethanol

In addition to the damage to DNA and histones caused by ethanol-induced ROS, ethanol neurotoxicity itself also has the same effect [7, 22, 27]. Regarding the view, Amanjot Kaur Riar et al. found that after exposure of alcohol to immature neurobasal progenitor cells, these cells were arrested in G1 phase and the percentage of S-phase cells markedly decreased [87]. In the subsequent study, they revealed that this alteration of cell cycle was associated with reduced levels of phosphorylation of cell cycle-regulatory proteins [87]. In particular, ethanol can directly damage DNA and increase its methylation. Likewise, the addition of alcohol to neuron culture system also results in different degrees of DNA double-strand breaks [88]. Strikingly, histones together with DNA to constitute chromosomes, are also subjected to ethanol attack. Moreover, through evaluation of PAE mice using partial wave spectroscopy (PWS) and confocal imaging with molecular-specific inverse participation ratios (IPR), it was found that the level of histone in the pup brains was remarkably reduced and their perimetry was also accelerated. The altered histone levels likely interfere with histone deposition and chromatin stability, thus resulting in dysregulation of gene expression [89, 90]. In addition, Ana M Romero identified an increased immunoreactive response to histone γH2AX, a DNA damage marker, and found that H2AX phosphorylation also prevented DNA damage foci from recruiting repair factor 53BP1 [88]. Therefore, these changes in histones are mainly attributed to a cascade reaction due to DNA methylation rather than direct effects of alcohol.

The alcoholic neurotoxic damage to DNA is frequently manifested in increased methylation of the corresponding genes. And beyond that, neuroinflammation causes a certain degree of these epigenetic dysregulation manifestations [25, 91, 92]. Current experimental animal studies have identified elevated DNA methylation due to PAE in protocadherins, glutamatergic synapses, and Hippo signaling-related genes in different embryo cerebral cortex of embryo [91–93]. In a clinical trial, Dipak K Sarkar also found increased methylation of proopiomelanocortin (POMC) and period 2 (PER2) genes in patients with FASD [94]. Notably, there is likely to be gender differences in epigenetic alterations, as demonstrated by higher male fetal methylation in pregnant women with PAE (1.5%; *p* = 0.01) [95]. Although several studies have found the phenomenon, a meta-analysis last year noted insufficient evidence in human and animal to support PAE as a cause of DNA methylation. This is likely attributed to heterogeneity and potential bias in different studies [96]. Therefore, a better-designed research is needed in the future to investigate the relationship between PAE and elevated DNA methylation.

### Impairment of physiological pathway by ethanol

The cell nucleus is particularly vulnerable to the alcoholic toxicity through a variety of patterns including intracellular metabolism, trophic cascades and signaling pathways [7, 22, 27, 28]. The administration of low- to medium-dose of ethanol promotes the expression of fibroblast growth factor 2 (FGF2) and its receptor in newborn mice [97]. The upregulation of FGF2-FGFR1 is usually accompanied by the changes of their downstream signaling molecules orexin and melanin-concentrating hormone (MCH) neuropeptides, which result in ectopic expression and obvious morphological changes of neurons, a hallmark of FASD occurrence [97, 98]. In addition, the effect of ethanolic toxicity on multiple trophic pathways in the embryo have been identified in PAE mice in multiple studies by Mauro Ceccanti, Fiore M et al. Among them, some neural associated neural-associated trophic signaling pathways are involved [99, 100]. The most susceptible members are the nerve growth factor (NGF) and brain-derived neurotrophic factor (BDNF) pathways, the suppression of these pathways markedly enormously reduced or altered the average speed of embryonic neural development [99–101]. Apart from the neurotrophic family, the ethanolic neurotoxicity involves insulin-like growth factor (IGF)-related pathways [23, 24]. Regarding the point, as early as 25 years ago, N Fatayerji found that PAE altered insulin-like growth factor binding protein (IGFBP) mRNA in embryonic mice [102]. The impact of ethanol on the IGF pathway is mainly due to alcoholic damage to G protein-coupled receptors and abnormal activation of various signaling cascades and phosphatases in tyrosine kinase receptors, which in turn inhibits IG-mediated neuronal development and movements of cerebellar granule neurons [23, 24, 103]. By interfering with the cAMP/PKA/EGR1 pathway, the vulnerability of neurons to ethanol drastically increases, thereby exacerbating neuronal destruction, which further triggers intrauterine growth restriction [104, 105]. Besides, alcohol can also disrupt embryonic brain development by dysregulating miR-145, NF-κB and Erk signaling pathways [43, 106].

In addition, alcoholic damage to the brain is hitherto found mainly in the hippocampus and prefrontal cortex [107, 108]. It is widely accepted notion that neuronal apoptosis and degeneration, imbalance neuronal excitation-inhibition and DNA hypermethylation damage are changes implicated in alcoholic damage in the hippocampal gateway, granule cell layer, CA1 and CA3 regions [107, 109]. This is likely to be associated with or even dependent on increased aquaporin-4 and neuroglial swelling [110]. Similarly, the hypermethylation also occurs in the prefrontal cortex (PFC) of the embryonic brain, and a significant increase in fragility of apoptotic cells and DNA damage rates in the PFC is found [108, 111]. Moreover, Nicholas A Heroux confirmed this view by investigating PAE rats and found that the immediate-early genes c-Fos, Arc, Egr-1 and Npas4 in the PFC regions were all functionally impaired [112]. Based on these results, alcoholic damage to the CNS affects neural development and functionality mainly through impairment of signaling pathways.

### Multiple patterns of alcohol damage to embryonic neuronal development

**Fig. 3:**
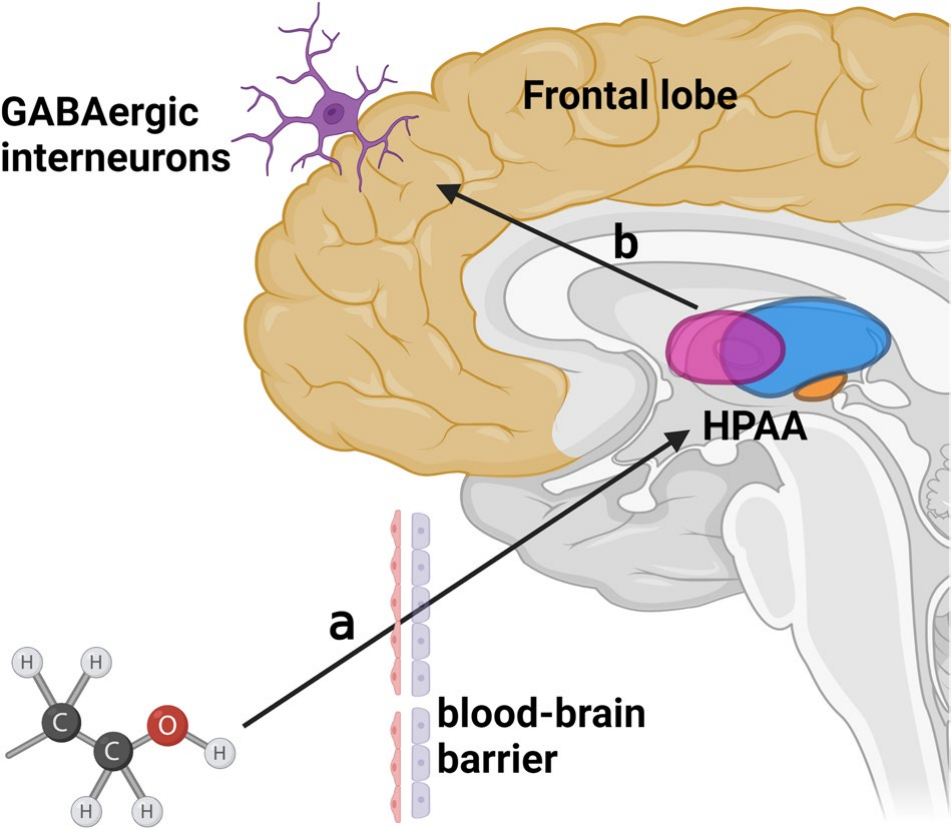
Ethanol causes migration and ectopia of inhibitory neurons. **a** Ethanol breaks blood–brain barrier to enter embryonic brain; **b** Ethanol hypersensitizes the HPAA axis and causes the generation of GABA interneurons and ectopic to the frontal cortex

The ethanol toxicity on the embryonic development of the nervous system often invokes several distinct neurological mechanisms apart from the damaging attack of genetic matters and various nutritional signaling pathways. Recent studies show that neurotoxic signaling cascades initiated by ethanol driving consequent abnormal emotion and behaviors have been linked to gamma-aminobutyric acid (GABA)-ergic neuronal injury in vulnerable brain areas. Given that GABAergic interneurons emerge in mid-gestation and are essential for the control of excitability and cognitive formation in cortical circuits, it is very critical to elucidate molecular mechanism of ethanol-induced toxicity to GABAergic neurons for preventing cortical circuit dysfunction and cognitive deficits in mid-gestation [113, 114]. Among PAE rats, ethanol causes hypersensitivity of the thalamus-pituitary-adrenal axis (HPAA) accompanied by demethylation of the glutamic acid decarboxylase 67 (GAD67) promoter, both of which result in a decrease in glutamatergic neurons and an increase in GABAergic neurons [26, 115]. Notably, Alexander G J Skorput further found that the increase in ectopic GABAergic interneurons after prenatal alcohol exposure was accompanied by migration [116, 117]. The GABA-expressing interneurons is ectopic from the ventral telencephalic proliferative zone to the prefrontal cortex and there is a selective decrease in the number of species, and the calmodulin (CR) and small albumin (PV) isoform counts show a significant decrease of > 30% in the neuron count [118, 119] (Fig. 3). These results were also confirmed by Florent Marguet in a clinical sampling for the patient study [120], suggesting that alcohol increases the quantity of the inhibitory neurotransmitters that hinder neuronal growth in the developing brain. The detailed mechanism of the neurotoxic effect of alcohol on GABAergic interneurons needs to be further investigated.

Extracellular molecules such as neural adhesion molecules (NCAM) are also attacked by ethanol, and alcohol inhibits its role in neuronal adhesion migration, differentiation [121, 122]. Multiple studies have demonstrated that ethanol could promote L1 coupling to the anchorin-G and hemosiderin-actin cytoskeletons by elevating phosphorylation of S1152, Y1176, S1181, and S1248, rendering L1 sensitive to ethanol [123, 124]. Concomitantly, ethanol binds with the Ig1-4 structural domain of L1 molecule and interferes with its physiological function [121, 122]. Additionally, ethanol disrupts the blood–brain barrier, which is thought to be the interface of neurotoxicity, it aggravates neurological disorders such as FASD [125]. Meanwhile, microglia are involved in ethanol-induced apoptosis of β-endorphin neurons by releasing vesicular exosomes in addition to stabilizing their M1 activation state during neuroinflammation [126, 127]. These studies are indicative of the diversity of alcohol-induced damage to embryonic neuronal development via distinct neurotoxic signaling cascades. Neurotoxic injury through multiple molecular mechanisms is summarized in Table 2

**Table 2:** The summary of oxidative stress injury through multiple molecular mechanisms

**Neurotoxic injury**	**Authors**	**Methods**		**Main findings**
**Epigenetic injury**	Ana M Romero et. Prakash A et. Nadia R et	C57BL/6 mice were exposed to 2.5 g/kg/d of 10% alcohol solution throughout pregnancy and labeled with ^2^H_2_O. Histones were analyzed using GC–MS, confocal	**Histone injury**	Alcohol causes deformation and reduction of histones in the embryonic brain and prevents the recruitment of repair factor 53BP1
	Sarkar DK et Loke YJ et Ozturk NC et Legault LM et	C57BL/6 mice were dosed with 2.5 g/kg ethanol given twice via injection in early to mid-pregnancy. Gene methylation in embryos was measured by MeCP2, qPCR. Clinical study, DNA methylation analysis of placenta, blood	**DNA hypermethylation**	DNA methylation is elevated in several signaling genes including POMC, PER2, calcitoninogen, glutamatergic synapses and Hippo
**Neurotrophic**	Chang GQ et Ceccanti M et	Mid-pregnancy rats were given 2 g/kg of ethanol (30%) orally. FGF2-FGFR1 and MCH were analyzed by RNA in situ hybridization, immunofluorescence C57BL/6 were orally treated with 1.5 g/kg of ethanol throughout the gestation. NGF and BDNF were quantitatively localized	**FGF2** **NGF, BDNF**	Ethanol causes abnormal MCH expression through activation of FGF2-FGFR1 NGF and BDNF are most sensitive to ethanol stimulation and significantly reduced
	Yu L, Zhou J et. Louis LK et	Human embryonic stem cells were exposed to 100 mM ethanol for 48 h. Proliferation and differentiation capacity and were subjected to whole genome analysis	**signal**	Ethanol intervention in neurodevelopment involves pathways of cAMP/PKA/EGR1, miR145, NF-κB and Erk
**Other pathways**	He X et. Leger C et. Skorput AG et	Wild-type and tPA knockout C57BL/6 mice were given 3 g/kg ethanol subcutaneously during mid- to late-pregnancy, while microglia cell were cultured (ethanol 1 mg/ml for 6 h)	**GABA**	Ethanol activates HPA axis, which causes a decrease in GABAergic neurons, meanwhile increases GABAergic interneuron expression and ectopia
	Dou X et	Intervention of NIH/3T3 clones stably expressing human hL1, analyzed by transfection silencing and flow cytometry assays	**NCAM**	Coupling of ethanol to anchoring-G and heme-actin cytoskeleton to enhance inhibition of NCAM

### Final outcome: neuronal damage and apoptosis

In spite of the modality, the ultimate outcome of alcohol damage to trophoblast cells almost is cell destruction or apoptosis [128], and the damaging neurotoxic effects of alcohol are immediate and long-lasting. In alcohol-exposed trophoblasts and neuronal cells, the number of surviving cells and total protein level markedly reduced and the apoptotic markers, like P-H2AX, caspase-3 and PARP-1 and BAX et al, increased [128, 129]. In addition, ethanol caused multiple disruption events in the neural crest region in PAE embryos, including intracellular calcium mobilization, elevated ROS, loss of β-linked protein/calcium, activation of mTOR signaling and others [130].

**Fig. 4:**
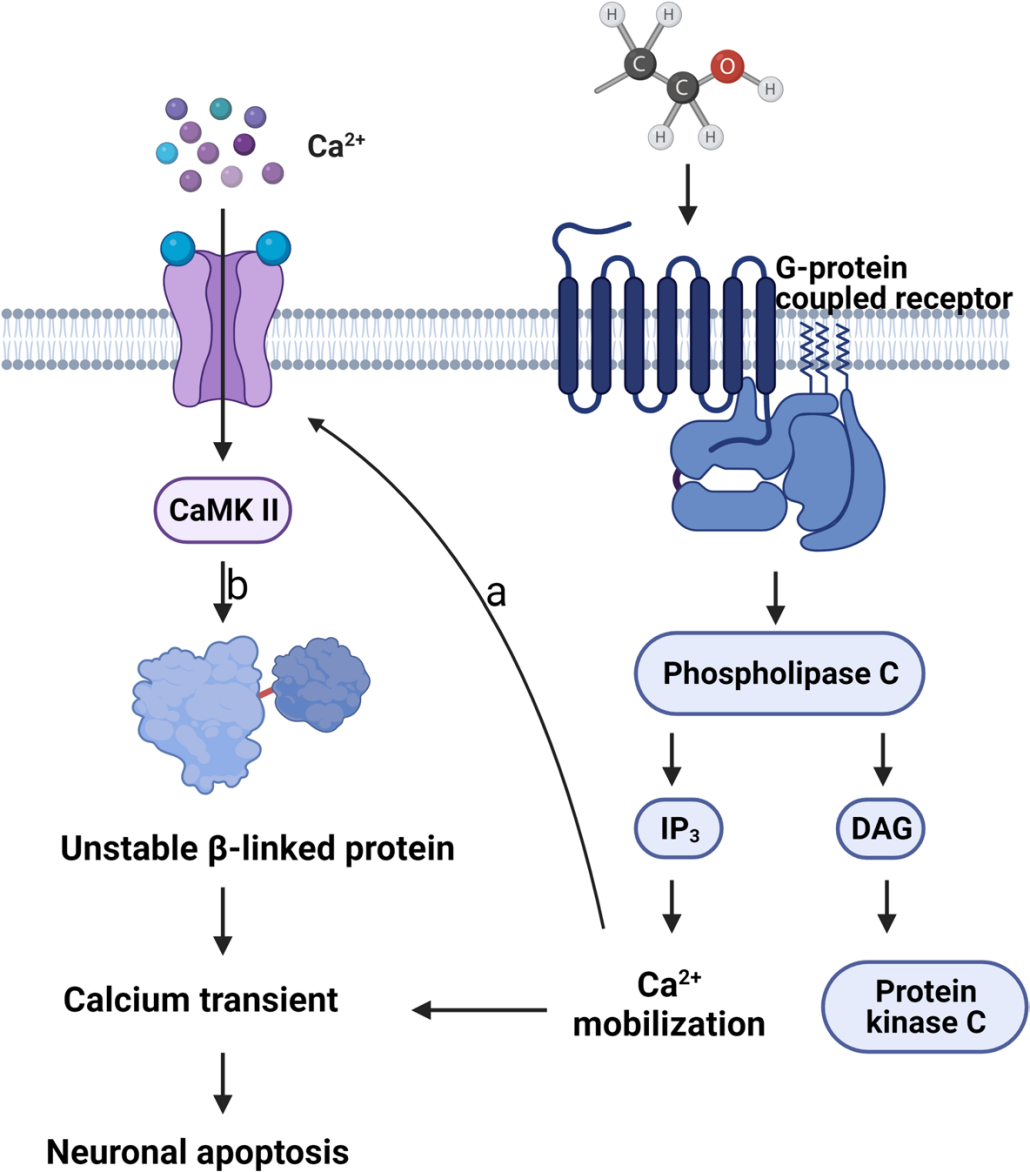
Ethanol elicits neuronal apoptosis through regulating calcium mobilization and G protein-coupled receptor driving cascades. **a** Calcium mobilization promotes cellular recruitment of extracellular Ca2 + ; **b** CaMKII hyperphosphorylation and activation leads to phosphorylated β-linked proteins, reducing their stability

Regarding the diversity of molecular mechanism underlying alcohol-induced neuronal damage and necrosis, A more clearly studied pathway to mediate cell apoptosis is to activate CaMKII through calcium mobilization [131, 132]. This apoptotic pathway is widely present in a variety of species [133]. In chick embryo experiments, alcohol exposure mobilized intra- and extracellular Ca^2+^ signaling, followed by selective and rapid enrichment of CaMKII [131, 132]. The critical signal, CaMKII, transforms the ethanol-induced, transitory Ca(i)(^2+^) into a long-lasting cellular effector [131]. The transduction of this signal belongs to G protein-dependent cGMP pathway (Fig. 4). Activation of CaMKII occurs by Ca^2+^/CaM binding to the regulatory domain, thereby opening the catalytic domain and making ATP binding β-linked protein a phosphorylated target [134, 135]. Subsequently, the stability and transcriptional activity of β-linked proteins are depressed, thereby initiating the neuron apoptosis and causing disease [136]. Regarding this notion, Ana Garic, Bolnick JM showed that cell apoptosis can be effectively inhibited through blockage of Ca^2+^ signaling by administration of CaMKII inhibitors to neurons in alcohol exposure, which provides more evidence that alcohol abuse leads to cell death by triggering the CaMKII pathway [131, 132]. Additionally, Flentke GR also reported that ethanol could antagonize the Snai2/p53 regulatory loop via Bcl-2 pathway, which suppresses the anti-apoptotic, pro-migratory and protective effect of the transcriptional repressor Snai [137]. In short, alcohol disrupts the regulatory loop between Snai2 and p53, leading to cell apoptosis.

### Current study status of FASD

#### Current knowledge of FASD

Almost all people are believed the harmful effects of alcohol abuse [2]. Given a general trend of increasing alcohol consumption worldwide, more and more harmful alcohol dependence and use seems to have become a certainty [1]. This is one of critical reasons why FASD is emerging as a major public health problem worldwide. Although not very high global prevalence of FASD has been reported, it is progressively increasing. Reportedly, multiple meta-analyses for past 5 years indicated that the global prevalence of FASD was approximately 7.7 per 1000 (95% CI, 4.9–11.7 per 1000). Averagely, every 67 women who consumed alcohol during pregnancy give birth to a FAS child [3, 8]. In recent years, the prevalence of FASD in 76 countries and territories exceeds FASD > 1% [19]. In addition, the incidence of FASD exists a significant difference between developed and developing regions. Through the study of FASD in the England and Canada, the prevalence of FASD is roughly estimated to reach approximately 2–3% among children screened in primary and community [9, 138]. In the Rocky Mountain region of the United States, this figure exhibits remarkable high percentage, ranging from 3.5–8.3% [139]. However, in the Eastern Mediterranean region this prevalence was only 0.1 per 1,000 population (95% CI, 0.1–0.5 per 1,000 population) [8]. Although the probability of FASD to FAS is currently less than 10%, FASD can also bring a diversity of lifelong physical and mental sufferings to patients themselves [16]. These data implicate the need for continuous attention to FASD and the great importance of social support and assistance to reduce the difficulties and sufferings faced by people with FASD [16, 20].

Combining long-term multiple investigations mentioned above, it could be inferred that there are primarily two pathways involved in the molecular mechanism of alcohol exposure leading to FASD, that is to say, neuroinflammation caused by metabolic ROS and the neurotoxicity of ethanol itself. The mechanism of neuroinflammatory damage caused by the metabolism of intake of ethanol resulting in excessive ROS that disrupts the oxidative metabolic balance in the embryo is well established [21, 45, 51]. This is also identified as the main mechanism. Neurotoxicity, in contrast, is a broad concept that encompasses damage from pathways other than oxidative stress. In summary, the mechanism by which PAE causes FASD in embryos is a multisystem, multifaceted, synergistic, persistent and lifelong effect, thus mediating neurodevelopmental impairment in the brain [7, 28, 29].

The above-mentioned neurotoxic damage is actually a broad concept. In addition to inflammation, ethanol can cause a variety of neuronal epigenetic variations, disruption of neural adhesion factors, blockage of physiological metabolic pathways, and structural changes in the brain. Still, the exact mechanism of the damage is still unclear. Accordingly, the multiple mechanisms of injury other than the inflammatory damage of ethanol that have been identified are collectively called as neurotoxic mechanisms. This is an area that will need to be further explored in the future. Regardless of the option to cause the injury, the outcome is neuronal damage and apoptosis, which leads to FASD.

### Current limitations and needs

Although the pathogenesis of FASD has become the topic of public interest and concern of numerous studies, a number of factors related to the pathogenesis of FASD other than neuroinflammation are still largely unresolved, and the mechanism is not fully understood. Accordingly, the study in this aspect will need to be further explored in the future. We refer to these pathways collectively as the neurotoxic damage of ethanol, which is the objective of this review.

Among a multitude of studies of PAE, more controversial is the establishment of animal models of PAE. Reversely, the use of different doses of ethanol exposure to simulate different levels of PAE is uncontroversial. Nevertheless, more controversial is still the timing of embryonic alcohol exposure leading to PAE. At present, the timing of embryonic alcohol exposure for the development of PAE includes maternal drinking at the beginning of gestation, 5–10 days of gestation, 12 days of gestation, and postnatal [64, 80, 117]. This may be useful to study the effects of alcohol exposure on the embryo at different gestational periods. On the other hand, different periods of alcohol exposure correspond to different periods of embryonic neurodevelopment. Alcohol exposure at different periods facilitates the study of subtypes of cells/molecules that develop significantly at a given stage and the effects on the embryo. Nevertheless, the criterion is still needed to assess the success of the establishment of a correct animal model of FASD.

The ultimate objective of further investigation of FASD's pathophysiology is to effectively aid in the early diagnosis and treatment. Currently, the FASD/FAS diagnostic guidelines have been gradually accepted by the medical community after several modifications. The diagnostic criteria for FAS must meet the following four requirements at the same time: unique physical traits (facial deformities); prenatal or postpartum development problems; neurological abnormalities; and aberrant mental and behavioral functioning [6, 140]. However, when FASD is definitively diagnosed, it has progressed to irreversible consequences or permanent birth defects. Since maternal self-reported drinking during pregnancy serves as a gold standard for early diagnosis, which clearly lacks objectivity and uncertainty [141]. Of the 222 children classified as having FASD, only two (< 1%) had documented a definite record of previous diagnosis of FASD [142]. Also, in the ascertainment study by Robyn McCarthy et al., only 3.6% of children with a 1.8% prevalence in Manchester had a definitive early diagnosis [9]. Besides self-reported alcohol consumption by pregnant women, a sensitive and specific biomarker and scale are required for the early diagnosis of FAS and FASD. These will facilitate implementing prevention, diagnosis, prevention, treatment, and management plans in time [143]. With further exploration of FASD, more and more physiological/pathological indicators of early changes such as fatty acid ethyl esters (FAEE) are being identified and used [144].

Despite the fact that people are raising awareness about the harmful effects of prenatal exposure to alcohol, alcohol consumption even alcohol dependence is still increasing rapidly every year [1]. Undoubtedly, the most effective strategy for both prevention and treatment of FASD is to abstain from alcohol before and during pregnancy [145]. Therefore, the future treatment should strive to reverse the progression of FASD while reducing or eliminating alcohol use. Currently, through further understanding the mechanism underlying FASD, several therapeutic options for FASD have emerged. Antioxidants could be used to counteract ethanol neuroinflammation, and reduce the damages caused by ROS. Notably, antioxidants such as folic acid has been proven to provide a therapeutic effect on FASD [146]. In addition, the supplement of some neuropeptides and trophic factors compensates for neuronal developmental delay resulting from disruption of trophic signaling pathways [101, 147]. Among the numerous treatment options, supplementation of Choline has generally been accepted for mitigating adverse effects of heavy drinking during pregnancy. Concurrently supplementation of Choline can repair and reverse the neuronal damage caused by ethanol, thus reducing the negative impact of fetal cognitive function. This view has been validated in both animal models and pre-clinical trials [148, 149].

The strength of this review is the systematic integration of recent research on the pathogenesis of FASD and indicative of currently needed research directions. However, despite the numerous studies related to FASD, there are still limitations in certain aspects. Among the more prominent are the differences in animal modeling criteria. Although the success of model construction can be identified by measuring embryonic blood alcohol concentration, it is possible that alcohol exposure during different gestational periods may lead to variability in neurological development, resulting in different experimental outcomes. In addition, there is still a lack of clear and effective diagnostic criteria and treatment protocols for the early diagnosis and treatment of FASD.

In the future, more studies are required to investigate the specific methods for detecting alcohol neurotoxicity on embryos and the underlying molecular mechanisms. This will provide a guidance in the early diagnosis and treatment of FASD. Given that our ultimate objective is to prevent and reduce the occurrence of FASD rather than a definitive diagnosis, children with FASD need more care and attention besides early clinical intervention. In addition, it is necessary to reduce the distress and stigma that FASD brings them and to solve the various inconveniences both in learning and neuropsychological problems.

## Conclusions

In summary, prenatal alcohol exposure to varying degree could cause excessive neuronal death and distinct impairments of the development of normal neural networks, leading to the pathogenesis of FASD. As a result, FASD causes medical, cognitive, behavioral, and social deficits that likely accompany throughout their lives. The neurological damage to the embryos caused by ethanol is multifaceted, multidirectional, persistent, and lifelong. Besides the damage caused by neuroinflammation and other related pathways are not yet fully understood, there are still many unresolved issues. For instance, the timing of embryonic alcohol exposure, beginning time of PAE, amount of intake alcohol and other complicated factors likely result in completely distinct clinical signs of FASD, which brings many difficulties to the diagnosis and treatment of the disease. Therefore, in-depth pre-clinical trials are still needed to scientifically elucidate the pathophysiology and pathogenesis of FASD by different animal models to develop ideal strategies to prevent, diagnose, and achieve breakthroughs of treatment of FASD. More importantly, education and prevention will need to be the most critical steps in avoiding FASD in the future.

## Data Availability

Not applicable.
